# Cell Cycle Genes Are Potential Diagnostic and Prognostic Biomarkers in Hepatocellular Carcinoma

**DOI:** 10.1155/2020/6206157

**Published:** 2020-06-07

**Authors:** Xu Liping, Li Jia, Chen Qi, Yang Liang, Li Dongen, Jiang Jianshuai

**Affiliations:** ^1^Department of Hepatobiliary Pancreatic Surgery, Ningbo First Hospital, Ningbo, Zhejiang Province, China; ^2^Department of Breast and Thyroid, Shanghai Tenth People's Hospital, Tongji University School of Medicine, Shanghai, China

## Abstract

**Background:**

The cell cycle pathway genes are comprised of 113 members which are critical to the maintenance of cell cycle and survival of tumor cells. This study was performed to investigate the diagnostic and prognostic values of cell cycle gene expression in hepatocellular carcinoma (HCC) patients.

**Methods:**

Clinical features and cell cycle pathway gene expression data were obtained from the Gene Expression Omnibus and The Cancer Genome Atlas databases. Differentially expressed genes (DEGs) were determined by the student *t*-test between HCC and noncancerous samples. Kaplan-Meier survival, univariate, and multivariate survival analyses and validation analysis were performed to characterize the associations between cell cycle gene expression and patients' overall survival and recurrence-free survival.

**Results:**

47 and 5 genes were significantly upregulated and downregulated genes in HCC samples, respectively. The high expression of *BUB3*, *CDK1*, and *CHEK1* was associated with increased mortality (adjusted *P* value = 0.04, odds ratio (OR): 1.89 (95% confidence interval (CI): 1.04-3.46); adjusted *P* value = 0.02, OR: 2.06 (95% CI:1.15-3.75); and adjusted *P* value = 0.04, OR: 1.84 (%95 CI: 1.03-3.32), respectively). The expression of *PTTG2* and *RAD21* was significantly associated with cancer recurrence (adjusted *P* value = 0.01, OR: 2.17 (95% CI: 1.24-3.86); adjusted *P* value = 0.03, OR: 1.88[95% CI:1.08-3.28], respectively), while the low expression of *MAD1L1* was associated with cancer recurrence (adjusted *P* value = 0.03, OR: 0.53 (%95 CI: 0.3-0.93)).

**Conclusions:**

The present study demonstrated that *BUB3*, *CDK1*, and *CHEK1* may serve as a prognostic biomarker for HCC patients. *PTTG2*, *RAD21*, and *MAD1L1* expression is a major factor affecting the recurrence of HCC patients.

## 1. Introduction

Liver cancer is the sixth most common cancer type and the fourth cause of cancer-associated mortalities in 2018 worldwide. Global cancer statistics shows that approximately 841,000 new liver cancer patients are diagnosed and 782,000 patients die of the disease annually [1]. Hepatocellular carcinoma (HCC) accounts for 75-85% of liver cancer cases and is the most common histological type of primary liver cancer. The major HCC-associated risk factors are hepatitis B and C virus infection, excessive alcohol drinking, exposure to aflatoxin, and smoking [2, 3]. Despite advances in the therapeutic methods, the 5-year overall survival (OS) rate is as low as 30% for the HCC patients who underwent surgical treatment [2, 4]. Therefore, the identification of more sensitive diagnostic and prognostic biomarkers is greatly important for the early diagnosis and improvement of prognosis in HCC patients.

Cell division consists of two consecutive processes, the interphase and mitosis. The interphase includes the G1 phase during which the cell prepares for DNA synthesis, S phase during which the replication of DNA occurs, and G2 phase during which the cell prepares for mitosis [5]. Cyclin-dependent kinases (CDKs) are key regulatory enzymes, each consisting of a catalytic CDK subunit and an activating cyclin subunit. CDKs regulate the cell's progression through the phases of the cell cycle by modulating the activity of key substrates. Downstream targets of CDKs include transcription factor E2F and its regulator Rb. Cell cycle deregulation associated with cancer occurs through the mutation of proteins important at different levels of the cell cycle. In cancer, mutations have been observed in genes encoding CDK, cyclins, CDK-activating enzymes, CKI, CDK substrates, and checkpoint proteins [6, 7].

The diagnostic and prognostic values of the cell cycle gene expression in HCC remain poorly understood. The primary objective of the present study was to investigate the cell cycle gene expression profile and the relationship between overall survival, recurrence-free survival (RFS), and cell cycle gene expression by analyzing a large set of HCC data from the Gene Expression Omnibus (GEO) [8, 9] and The Cancer Genome Atlas (TCGA) databases [10].

## 2. Methods and Materials

### 2.1. Data Acquisition

The clinical characteristics of HCC patients and expression data of cell cycle genes were obtained from the GSE14520 dataset of the GEO database (https://www.ncbi.nlm.nih.gov/geo/query/acc.cgi?acc=GSE14520) [8, 9]. The gene expression values of the GSE14520 dataset were robust multiarray average (RMA) normalized signal intensities. The clinical factors analyzed in this study included age, gender, serum *α*-fetoprotein (AFP) level, cirrhosis, main tumor size, multinodular tumors, tumor-node-metastasis (TNM) stage, survival time, survival status, time to recurrence, and recurrence status. The HCC cohort was downloaded from the TCGA database for the validation analysis [10]. The TCGA dataset included survival status, follow-up time, recurrence status, and time to recurrence of 377 HCC patients and cell cycle expression levels of 377 HCC patients and paired 50 noncancerous tissues. The expression values of the TCGA dataset were normalized read counts for 113 cell cycle genes. 113 genes in the cell cycle pathway were downloaded from the Kyoto Encyclopedia of Genes and Genomes (KEGG) database (https://www.genome.jp/dbget-bin/www_bget?pathway+hsa04110) [11]. As the datasets included in the study were downloaded from public databases, the study did not need the approval of an ethics committee.

### 2.2. Bioinformatics Analysis of Cell Cycle Genes

To investigate the biological functions and possible signaling pathways of cell cycle genes, the enrichment of gene ontology (GO) terms and KEGG pathways was analyzed by the Database for Annotation, Visualization, and Integrated Discovery (DAVID) bioinformatics online tool, version 6.8 (https://david.ncifcrf.gov/) [12].

### 2.3. Diagnostic Analyses of Cell Cycle Genes

Differentially expressed genes (DEGs) were determined by the student *t*-test between 247 HCC tissues and 241 noncancerous tissues. Raw *P* values were corrected by the Bonferroni method. The genes with adjusted *P* values < 0.05 were considered DEGs. The 50 pairs of HCC samples and noncancerous samples from the TCGA dataset were used to validate the DEGs between primary HCC tissues and normal liver tissues. Receiver operating characteristic (ROC) curve analysis was conducted by the R package of pROC to determine the diagnostic values of the differentially expressed genes [13]. Area under the curve (AUC) values were computed accordingly by the R package of pROC for cell cycle genes.

### 2.4. Survival and Recurrence Analyses

The Fisher exact test was used to analyze the associations between overall survival and clinical factors, including age, gender, serum AFP level, cirrhosis, main tumor size, multinodular tumors, and TNM stage, in the GEO dataset. To characterize the associations of the cell cycle gene expression with patients' overall survival, HCC patients were divided into the “high-expression” or “low-expression” group if they exhibited expression levels greater or smaller than the median values, respectively. Kaplan-Meier (KM) survival analysis was performed to plot survival curves, and the log-rank test was utilized to compare the difference in survival rates between the high- and low-expression groups using the R package of survival [14]. Univariate and multivariate survival analyses were performed using the logistic regression model. *P* < 0.05 was considered statistically significant. The methods for recurrence-free survival analyses were the same with overall survival analyses.

### 2.5. Prognostic Nomogram for Survival Prediction

All 247 HCC patients in the GSE14520 dataset were used for nomogram construction. The prognosis-associated clinical variables were selected to develop the overall survival nomogram, including tumor size, multinodular, cirrhosis, serum AFP level, TNM stage, and the *BUB3*, *CDK1*, and *CHEK1* expression. The recurrence-associated clinical factors, sex, TNM stage, and the *PTTG2*, *RAD21*, and *MAD1L1* expression were included in the recurrence nomogram. The nomograms were constructed by the R package of rms (v5.1-3.1). Each variable was assigned a score, and the scores of all variables were summed to calculate the total point, which was located onto the scale. Thus, the probabilities of the survival outcome could be predicted by drawing a vertical line to the total point.

## 3. Results

### 3.1. Characteristics of Patients in the GEO Database

The GSE14520 dataset of the GEO database has 247 patients available for survival analysis. Detailed characteristics of these patients are shown in Table 1. Tumor size, multinodular, cirrhosis, serum AFP level, and TNM stage were found to be positively associated with OS (*P* < 0.05 for all cases, Fisher's exact test, Table 1), whereas sex and TNM stage were positively associated with cancer recurrence (*P* values < 0.05 for all cases, Fisher's exact test, Table 1). The remaining characteristics did not exhibit a significant association with OS or cancer recurrence (*P* values > 0.05 for all cases, Fisher's exact test, Table 1).

### 3.2. Bioinformatics Analysis of Cell Cycle Genes

The GO function analysis indicated that cell cycle genes were mainly enriched in the regulation of cell division, mitotic nuclear division, G1/S transition of mitotic cell cycle, and DNA replication (Supplementary Table 1, adjusted *P* values < 0.05 for all cases). The KEGG pathway analysis suggested that cell cycle genes were associated with the WNT signaling pathway, chronic myeloid leukemia, pathways in cancer, and other pathways (Supplementary Table 2, adjusted *P* values < 0.05 for all cases).

### 3.3. Assessment of Diagnostic Value

**Figure 1:**
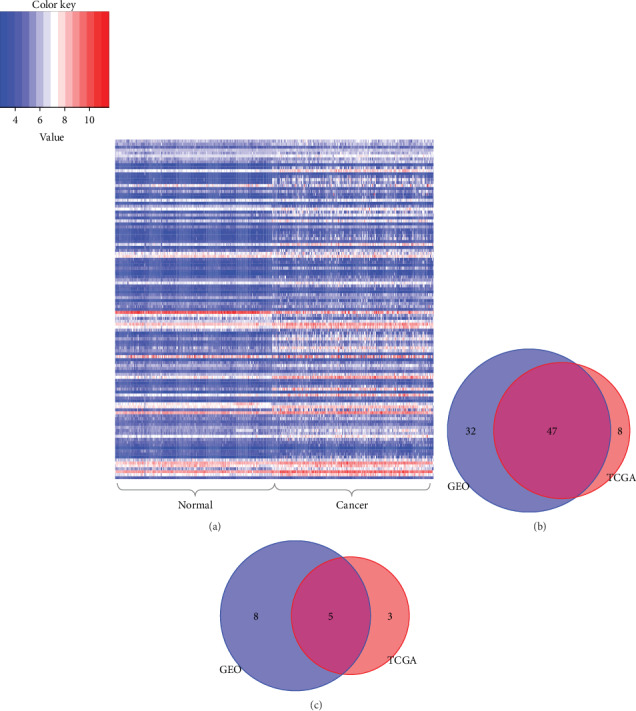
Differentially expressed gene analysis. (a). The 92 DEGs between HCC samples and noncancerous samples in the GEO dataset. The gene expression values are a robust multiarray average (RMA) normalized signal intensity on the color scale. (b) The overlap of upregulated genes between the TCGA and GEO datasets. (c) The overlap of downregulated genes between the TCGA and GEO datasets.

**Figure 2:**
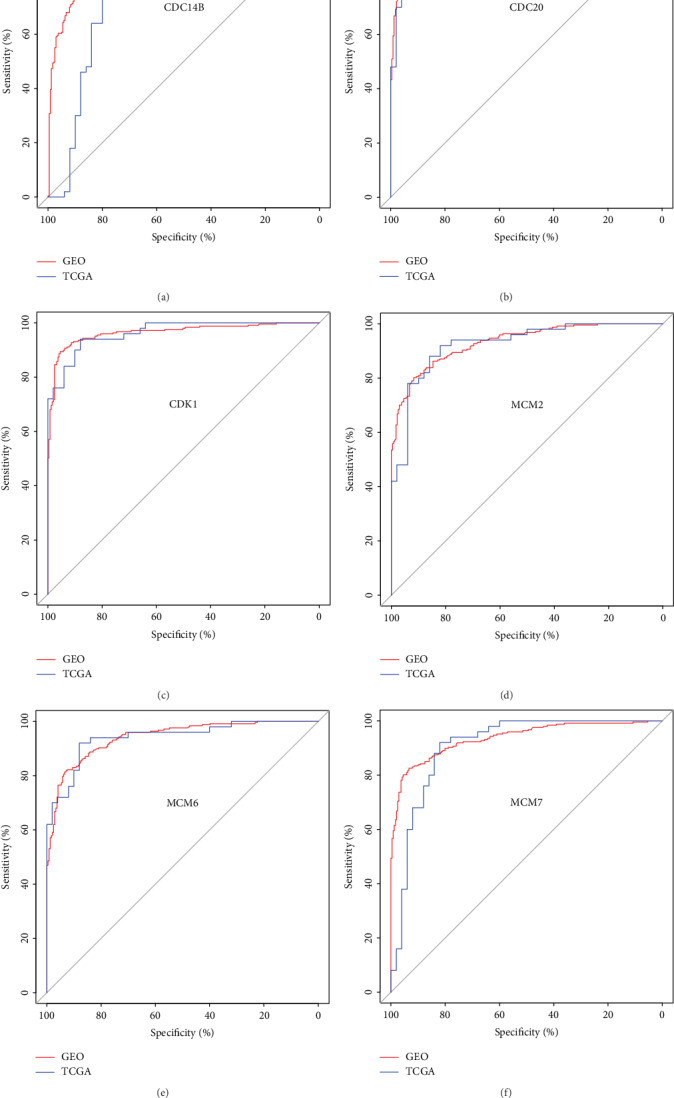
The receiver operating characteristics (ROC) curves of CDC14B (a), CDC20 (b), CDK1 (c), MCM2 (d), MCM6(e), and MCM7(f) in distinguished HCC tumor tissues and adjacent nontumor tissues in the GEO and TCGA datasets.

By comparing cell cycle gene expression levels between 247 tumor tissues and 241 adjacent nontumor tissues, 92 cell cycle genes were found to be differentially expressed between tumor and nontumor tissues. The DEGs included 79 upregulated and 13 downregulated genes in HCC samples (Figure 1(a) and Supplementary Table 3, adjusted *P* values < 0.05 for all cases). The TCGA dataset was used to further validate the cell cycle genes that were differentially expressed between normal liver tissues and primary HCC tissues. The validation analysis confirmed that 47 and 5 genes were significantly upregulated and downregulated genes in HCC samples (Figures 1(b) and 1(c), adjusted *P* values < 0.05 for all cases). ROC curves were constructed to further explore the diagnostic values of these 52 DEGs. 30 cell cycle genes had a potential prediction value, with all *P* values < 0.05 and AUC > 0.8 for the GEO and TCGA datasets (Supplementary Table 5); *CDC14B*, *CDC20*, *CDK1*, *MCM2*, *MCM6*, and *MCM7* in particular exhibited high accuracy in differentiating HCC tissues from nontumor tissues (Figure 2, *P* values < 0.05, AUC > 0.8 for all cases).

### 3.4. Survival Analysis

**Figure 3:**
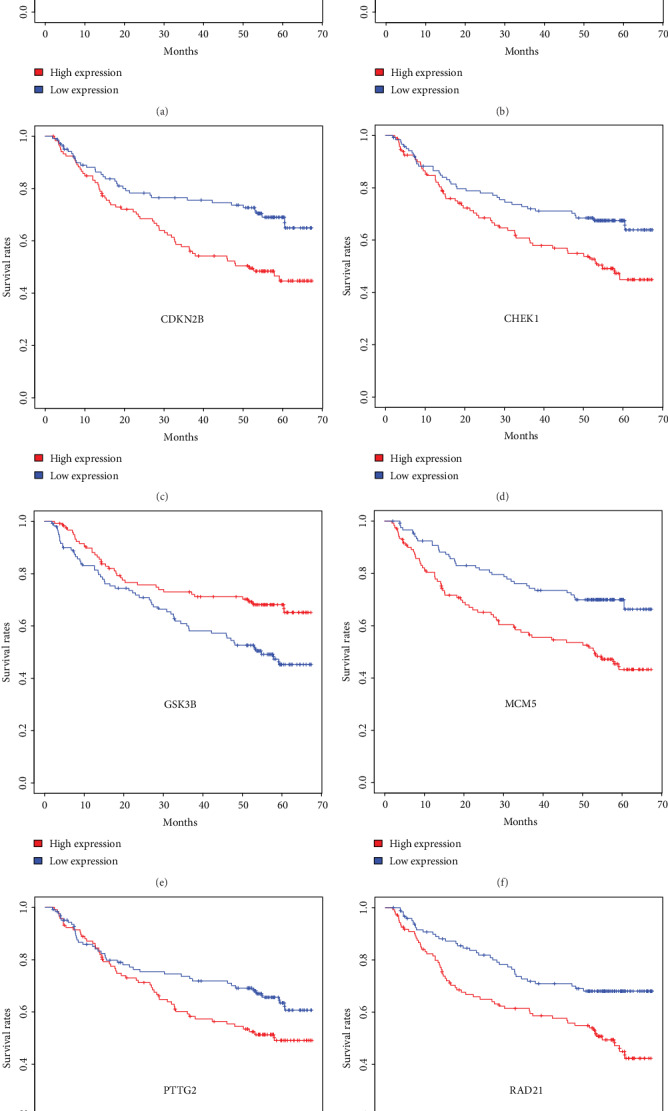
The Kaplan-Meier survival analysis results of BUB3 (a), CDK1 (b), CDKN2B (c), CHEK1 (d), GSK3B (e), MCM5 (f), PTTG2 (g), and RAD21 (h) in the GEO dataset.

To evaluate the predictive capability of cell cycle gene expression for patients' overall survival, the 247 HCC patients were divided into the low- and high-expression groups based on median values. Kaplan-Meier survival analysis suggested that the high expression of 30 genes and low expression of 5 genes were associated with a poor overall survival (*P* < 0.05 for all cases, log-rank test, Supplementary Table 6). Univariate analysis using the logistic regression model showed that the high expression of 21 genes was significantly associated with increased mortality, while the high expression of 2 genes was associated with decreased mortality (*P* < 0.05 for all cases, Supplementary Table 6). The characteristics associated with clinical prognostic outcome, including tumor size, multinodular, cirrhosis, serum AFP level, and tumor stage, were included in the multivariate cox regression analysis. Following the adjustment of the prognosis-related risk factors, the expression of 8 genes was significantly associated with OS in the survival analysis (adjusted *P* values < 0.05 for all cases, Table 2 and Figure 3). The high expression of *BUB3*, *CDK1*, *CDKN2B*, *CHEK1*, *MCM5*, *PTTG2*, and *RAD21* was associated with increased mortality (adjusted *P* value = 0.04, odds ratio (OR): 1.89 (95% confidence interval (CI): 1.04-3.46); adjusted *P* value = 0.02, OR: 2.06 (95% CI:1.15-3.75); adjusted *P* value = 0.00, OR: 2.92 (95% CI:1.61-5.41); adjusted *P* value = 0.04, OR: 1.84 (%95 CI: 1.03-3.32); adjusted *P* value = 0.01, OR: 2.07 (%95 CI:1.16-3.72); adjusted *P* value = 0.04, OR: 1.87 (%95 CI:1.03-3.44); and adjusted *P* value = 0.00, OR: 2.49 (%95 CI:1.38-4.60), respectively), while the low expression of *GSK3B* was associated with increased mortality (adjusted *P* value = 0.04, OR: 0.55 (%95 CI: 0.3-0.78)). To further validate the associations between overall survival and the 8 genes above, the TCGA cohort was used to conduct the KM analysis. The survival analysis results suggested that *BUB3*, *CDK1*, and *CHEK1* were found to be associated with the OS of HCC patients in the TCGA cohort (*P* values < 0.05 for all cases, log-rank test, Supplementary Figure 1).

### 3.5. Recurrence-Free Survival Analysis

To analyze the associations of cell cycle gene expression with patients' RFS, the 247 HCC patients were divided into the low- and high-expression groups based on median values. Kaplan-Meier analysis suggested that the high expression of 9 genes, *CDKN1C*, *CDC25B*, *CDC20*, *PTTG2*, *SMC3*, *RAD21*, *EP300*, *CDC25A*, and *MCM5* and low expression of 3 genes, *MAD1L1*, *GADD45A*, and *GADD45G* were associated with RFS in HCC (*P* values < 0.05 for all cases, log-rank test, supplementary table 7). Univariate analysis using the logistic regression model showed that the high expression of *PTTG2* and *RAD21* was significantly associated with tumor relapse, while low expression of *MAD1L1* was associated with tumor relapse (*P* values < 0.05 for all cases, supplementary table 7). The characteristics associated with cancer recurrence, including gender and tumor stage, were included in the multivariate Cox regression analysis. Following the adjustment of the recurrence-related risk factors, the expression of *PTTG2* and *RAD21* was significantly associated with cancer recurrence (adjusted *P* value = 0.01, OR: 2.17 (95% CI: 1.24-3.86); adjusted *P* value = 0.03, OR: 1.88 (95% CI:1.08-3.28),respectively), while the low expression of *MAD1L1* was associated with cancer recurrence (adjusted *P* value = 0.03, OR: 0.53 (%95 CI: 0.3-0.93), supplementary table 7). The TCGA dataset was used to validate the associations of 12 cell cycle genes with RFS in HCC patients. The high expression of *CDC20*, *PTTG2*, and *CDC25A* was positively associated with cancer recurrence (*P* value = 0.02, OR: 1.72 (95% CI: 1.09-2.74); *P* value = 0.65, OR: 1.11 (95% CI: 0.7-1.76); and *P* value = 0.16, OR: 1.39 (95% CI: 0.88-2.2)), while the increased expression of *GADD45A* was negatively associated with cancer recurrence (*P* value = 0.99, OR: 1 (95% CI: 0.63-1.59), supplementary table 8).

### 3.6. Prognostic Nomogram for Survival Prediction

**Figure 4:**
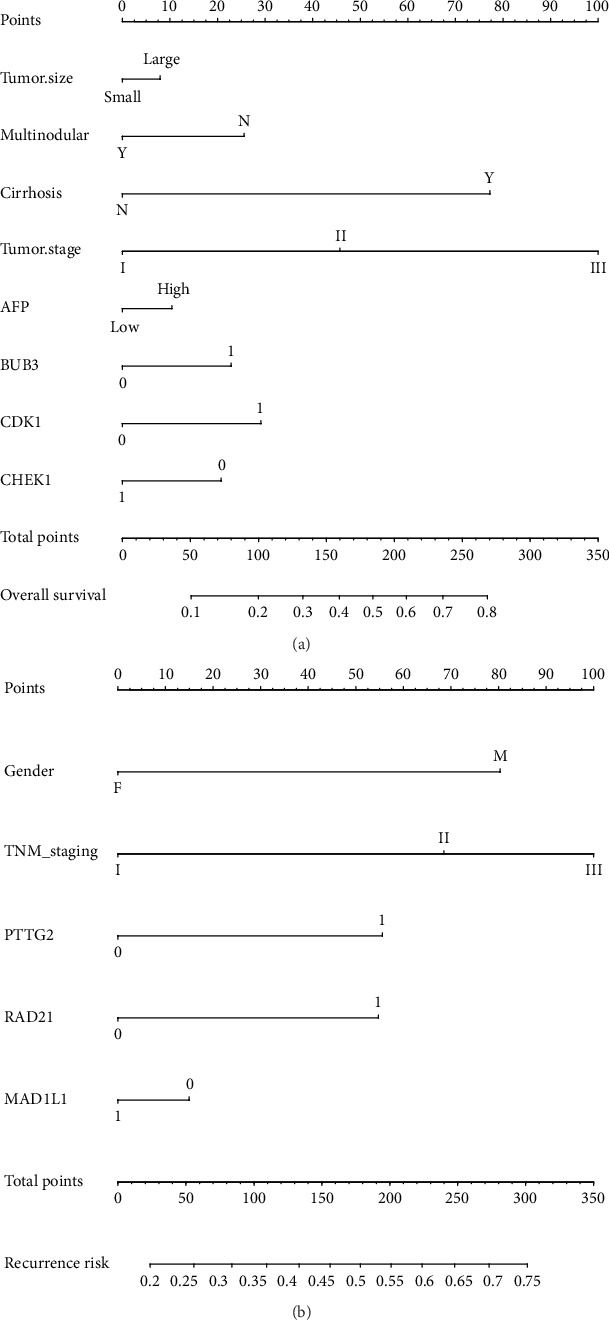
Prognostic nomogram for survival prediction. (a) Nomogram for overall survival. (b) Nomogram for recurrence-free survival.

The prognostic risk factors that may predict the outcome of survival, including sex, serum AFP level, cirrhosis, TNM stage, tumor size, and cell cycle gene expression, were selected to construct the nomogram, which can provide an individualized prognosis prediction. For the 247 HCC patients, nomogram analysis was performed for the probabilities of death (Figure 4(a)) and recurrence event (Figure 4(b)). As shown in the nomogram, *BUB3*, *CDK1*, and *CHEK1* expression contributed to a certain extent to the patients' overall survival and *PTTG2*, *RAD21*, and *MAD1L1* expression is a major factor affecting the RFS of HCC patients.

## 4. Discussion

The aim of the present study was to investigate the diagnostic and prognostic values of cell cycle gene expression in HCC patients by analyzing a large set of HCC data from the GEO and TCGA database. The results suggested that 30 genes may serve as potential diagnostic biomarkers for HCC patients with high accuracy. Some genes are known diagnostic biomarkers in cancers, such as *CDK1* and *CDC20*. High expression of *CDC20*, a key component of the spindle assembly checkpoint (SAC), has been reported in various malignancies, and *CDC20* plays a vital role in tumorigenesis and progression. *CDC20* is overexpressed in a wide range of tumor types, including prostate [15], bladder, cervix, liver, stomach, thyroid, and colon cancer [16–18]. High *CDC20* expression was associated with advanced tumor stage in breast, colon, endometrium, and prostate cancer [16, 18] and HCC [17]. In addition to the known DEGs, this study, for the first time, reported a set of new diagnostic biomarkers in HCC patients, such as *CDC14B*, *CCNE1*, *CCNE2*, and *CDKN2C*.

AFP shows a sensitivity of 39%– 65% and a specificity of 76%–94% in the screening for HCC, suggesting AFP as a screening tool might miss a large fraction of HCC patients [19]. In our study, we found that 30 genes effectively discriminated patients with HCC from healthy controls (AUC > 0.80 for all cases). The performance of these genes for HCC is superior to AFP and may provide a more cost-effective and less resource-intensive method. Prospective clinical evaluation is needed to compare or potentially combine AFP screening with these genes.

In addition, we demonstrated that the expression level of *BUB3*, *CDK1*, and *CHEK1* was significantly associated with mortality, with patients with a higher expression level of *BUB3*, *CDK1*, and *CHEK1* expected to have a poor prognostic outcome. *BUB3* is a member of the spindle assembly checkpoint genes which maintain accurate chromosomal segregation and prevents the formation of aneuploidy during mitosis. Germline mutations in *BUB1* and *BUB3* are associated with an increased risk of colorectal cancer [20]. *BUB3* expression is upregulated in oral squamous cell carcinoma patients. A high expression of *BUB3* was an independent prognostic indicator for cancer-specific survival and was associated with increased cellular proliferation [21]. There are few reports on the functions of *BUB3* in HCC patients; this study for the first time revealed that *BUB3* was a negative prognostic biomarker for HCC patients.

*CDK1* is a member of the Ser/Thr protein kinase family. This protein is a catalytic subunit of the highly conserved protein kinase complex known as the M-phase-promoting factor, which plays an important role in cell division [22]. In line with the findings in our study, *CDK1* is overexpressed in several cancer types, including laryngeal squamous cell carcinoma [23], lung cancer [24], HCC [25], epithelial ovarian cancer [26], and pancreatic ductal adenocarcinoma [27]. A high expression of *CDK1* is negatively associated with overall survival for lung cancer [24], HCC [28], epithelial ovarian cancer [26], and pancreatic ductal adenocarcinoma [27]. *CDK1* expression was significantly higher in the bone marrow from acute myeloid leukemia (AML) patients at recurrence than that at initial diagnosis. AML patients with higher level of nuclear *CDK1* in their leukemic blasts showed inferior clinical outcome compared with those with lower levels [29]. Additionally, *CDK1* modulates the levels of P27(kip) and AKT phosphorylation in response to all-trans retinoic acid treatment in AML patients. The regulation of the subcellular content of CDK1 and RAR*γ* by all-trans retinoic acid is an important process for achieving an effective response in treatment of leukemia [29].

*CHEK1* is another member of the Ser/Thr protein kinase family. It has crucial roles in the checkpoint-mediated cell cycle arrest in response to DNA damage or the presence of unreplicated DNA. As previously reported, *CHEK1*, both at the mRNA and protein levels, is highly expressed in medulloblastoma [30] and T-cell acute lymphoblastic leukemia [31]. Elevated *CHEK1* expression in medulloblastoma is an adverse prognostic marker [30]. Cytoplasmic expression of *CHEK1* was associated with higher grade, triple-negative phenotype, KI67, p53, AKT, and PI3K expression in breast cancer [32]. The function of *CHEK1* has been rarely reported in HCC; our study demonstrated that *CHEK1* might serve as an adverse prognostic biomarker for HCC patients.

*BUB3*, *CDK1*, and *CHEK1* expression profiling may guide the treatment for HCC patients in the clinical settings. If the specimens of HCC patients exhibit high *BUB3*, *CDK1*, and *CHEK1* expression, these patients are probably associated with an inferior prognosis. Therefore, these patients might need a more aggressive treatment or frequent follow-up. *BUB3*, *CDK1*, and *CHEK1* may also pave the way for developing targeted therapies for HCC patients. For instance, the *CDK1* inhibitor, purvalanol A, enhances the taxol-induced apoptosis and inhibitory effects on cellular proliferation of taxol through Op18/stathmin in non-small-cell lung cancer cells in vitro [33]. Knockdown of *CDK1* allowed cancer cells to undergo active mitosis and inhibited their sensitivity to all-trans retinoic acid-induced cell cycle arrest in AML cells [29].

## 5. Conclusion

Taken together, the results of the present study demonstrated that *CDC14B*, *CDC20*, *CDK1*, *MCM2*, *MCM6*, and *MCM7* may be potential diagnostic biomarkers and *BUB3*, *CDK1*, and *CHEK1* may serve as a negative prognostic biomarker for HCC patients. *PTTG2*, *RAD21*, and *MAD1L1* expression is a major factor affecting the recurrence of HCC patients.

## Figures and Tables

**Table 1:** Association between the clinicopathologic characteristics and overall survival status and recurrence status.

Variables		Alive	Dead	P value	Nonrecurrence	Recurrence	*P* value
Gender	Female	23	8	0.12	21	10	0.01
	Male	123	88		85	126	
Age	>45	99	67	0.78	71	95	0.68
	≤45	47	29		35	41	
ALT	>50 U/l	57	43	0.42	36	64	0.05
	≤50 U/l	89	53		70	72	
Tumor size	>5 cm	44	44	0.02	35	53	0.42
	≤5 cm	101	52		70	83	
Multinodular	No	122	68	0.02	87	103	0.27
	Yes	24	28		19	33	
Cirrhosis	No	17	2	0.01	12	7	0.09
	Yes	129	94		94	129	
Tumor stage	I	75	21	0.00	57	39	0.00
	II	46	32		28	50	
	III	18	33		15	36	
AFP	>300 ng/ml	58	52	0.03	44	66	0.36
	≤300 ng/ml	85	43		59	69	

**Table 2:** Survival analysis of 8 cell cycle genes in the GEO dataset.

Gene	Univariate analysis			Multivariate analysis	
	Median	OR (2.5%CI-97.5%CI)	*P* value	OR (2.5%CI-97.5%CI)	*P* value
*BUB3*	7.80	1.87 (1.11-3.17)	0.02	1.89 (1.04-3.46)	0.04
*CDK1*	5.35	2.16 (1.28-3.68)	0.00	2.06 (1.15-3.75)	0.02
*CDKN2B*	3.61	2.57 (1.52-4.40)	0.00	2.92 (1.61-5.41)	0.00
*CHEK1*	3.63	1.87 (1.11-3.17)	0.02	1.84 (1.03-3.32)	0.04
*GSK3B*	6.49	0.46 (0.27-0.78)	0.00	0.55 (0.3-0.78)	0.04
*MCM5*	5.49	2.32 (1.38-3.96)	0.00	2.07 (1.16-3.72)	0.01
*PTTG2*	3.22	1.82 (1.08-3.07)	0.02	1.87 (1.03-3.44)	0.04
*RAD21*	8.12	2.32 (1.38-3.96)	0.00	2.49 (1.38-4.60)	0.00

## Data Availability

The datasets used and/or analyzed during the current study are available from the corresponding author on reasonable request.

## Abbreviations

HCC: Hepatocellular carcinoma
GEO: Gene Expression Omnibus
TCGA: The Cancer Genome Atlas
OS: Overall survival
RFS: Recurrence-free survival
AFP: *α*-Fetoprotein
TNM: Tumor-node-metastasis
GO: Gene ontology
KEGG: Kyoto Encyclopedia of Genes and Genomes
DAVID: Database for Annotation, Visualization, and Integrated Discovery
DEGs: Differentially expressed genes
ROC: Receiver operating characteristic
AUC: Area under curve
OR: Odd ratio
CI: Confidence interval
KM: Kaplan-Meier
AML: Acute myeloid leukemia.

## Supplementary Materials

Supplementary Materials 1. Supplementary tables present the enrichment of GO terms, KEGG pathways of cell cycle genes, differentially expressed genes in the GEO and TCGA datasets, and the associations of cell cycle genes with overall survival and recurrence-free survival of HCC patients. 2. Supplementary Figure 1: the Kaplan-Meier survival analysis results of BUB3 (A), CDK1 (B), and CHEK1 (C) in the TCGA dataset.
