# Corrigendum to “Cell Cycle Genes Are Potential Diagnostic and Prognostic Biomarkers in Hepatocellular Carcinoma”

**DOI:** 10.1155/2020/8679532

**Published:** 2020-11-21

**Authors:** Xu Liping, Li Jia, Chen Qi, Yang Lian, Li Dongen, Jiang Jianshuai

**Affiliations:** ^1^Department of Hepatobiliary Pancreatic Surgery, Ningbo First Hospital, Ningbo, Zhejiang Province, China; ^2^Department of Breast and Thyroid, Shanghai Tenth People's Hospital, Tongji University School of Medicine, Shanghai, China

In the article titled “Cell Cycle Genes Are Potential Diagnostic and Prognostic Biomarkers in Hepatocellular Carcinoma” [1], information was omitted from Acknowledgments in error. Acknowledgments is shown below.
