# Supplementary material for: Cell Cycle Genes Are Potential Diagnostic and Prognostic Biomarkers in Hepatocellular Carcinoma
Source: Biomed Res Int. 2020 Jun 7;2020:6206157. doi: 10.1155/2020/6206157 (PMC7298261; doi:10.1155/2020/6206157)
Supplement: Supplementary Materials — 1. Supplementary tables present the enrichment of GO terms, KEGG pathways of cell cycle genes, differentially expressed genes in the GEO and TCGA datasets, and the associations of cell cycle genes with overall survival and recurrence-free survival of HCC patients. 2. Supplementary Figure 1: the Kaplan-Meier survival analysis results of BUB3 (A), CDK1 (B), and CHEK1 (C) in the TCGA dataset. [file 6206157.f1.docx]

Supplementary Table1. GO terms of cell cycle genes

| GO term | Term name | Gene count | Fold enrichment | P value | Benjamini-adjusted P value |
| --- | --- | --- | --- | --- | --- |
| GO:0051301 | cell division | 51 | 19.73 | 0.00 | 0.00 |
| GO:0007067 | mitotic nuclear division | 35 | 19.11 | 0.00 | 0.00 |
| GO:0000082 | G1/S transition of mitotic cell cycle | 37 | 49.12 | 0.00 | 0.00 |
| GO:0006270 | DNA replication initiation | 17 | 71.94 | 0.00 | 0.00 |
| GO:0006260 | DNA replication | 24 | 20.97 | 0.00 | 0.00 |
| GO:0051439 | regulation of ubiquitin-protein ligase activity involved in mitotic cell cycle | 17 | 100.09 | 0.00 | 0.00 |
| GO:0051437 | positive regulation of ubiquitin-protein ligase activity involved in regulation of mitotic cell cycle transition | 21 | 37.42 | 0.00 | 0.00 |
| GO:0031145 | anaphase-promoting complex-dependent catabolic process | 21 | 36.00 | 0.00 | 0.00 |
| GO:0051436 | negative regulation of ubiquitin-protein ligase activity involved in mitotic cell cycle | 19 | 36.24 | 0.00 | 0.00 |
| GO:0042787 | protein ubiquitination involved in ubiquitin-dependent protein catabolic process | 23 | 20.36 | 0.00 | 0.00 |
| GO:0070979 | protein K11-linked ubiquitination | 13 | 65.20 | 0.00 | 0.00 |
| GO:0043161 | proteasome-mediated ubiquitin-dependent protein catabolic process | 21 | 14.01 | 0.00 | 0.00 |
| GO:0007050 | cell cycle arrest | 19 | 18.25 | 0.00 | 0.00 |
| GO:0042326 | negative regulation of phosphorylation | 7 | 47.40 | 0.00 | 0.00 |
| GO:0008285 | negative regulation of cell proliferation | 17 | 5.81 | 0.00 | 0.00 |
| GO:0030308 | negative regulation of cell growth | 10 | 11.19 | 0.00 | 0.00 |
| GO:1900740 | positive regulation of protein insertion into mitochondrial membrane involved in apoptotic signaling pathway | 10 | 45.14 | 0.00 | 0.00 |
| GO:0061024 | membrane organization | 7 | 29.62 | 0.00 | 0.00 |
| GO:0006605 | protein targeting | 5 | 17.36 | 0.00 | 0.00 |
| GO:0090399 | replicative senescence | 7 | 78.99 | 0.00 | 0.00 |
| GO:1901796 | regulation of signal transduction by p53 class mediator | 10 | 10.92 | 0.00 | 0.00 |
| GO:0006975 | DNA damage induced protein phosphorylation | 4 | 67.71 | 0.00 | 0.00 |
| GO:0006974 | cellular response to DNA damage stimulus | 10 | 6.51 | 0.00 | 0.00 |
| GO:0008630 | intrinsic apoptotic signaling pathway in response to DNA damage | 6 | 17.29 | 0.00 | 0.00 |
| GO:0000077 | DNA damage checkpoint | 5 | 22.57 | 0.00 | 0.00 |
| GO:0071480 | cellular response to gamma radiation | 4 | 25.79 | 0.00 | 0.01 |
| GO:0090399 | replicative senescence | 7 | 78.99 | 0.00 | 0.00 |
| GO:0042771 | intrinsic apoptotic signaling pathway in response to DNA damage by p53 class mediator | 4 | 17.47 | 0.00 | 0.02 |
| GO:0000320 | re-entry into mitotic cell cycle | 3 | 101.56 | 0.00 | 0.00 |
| GO:0010718 | positive regulation of epithelial to mesenchymal transition | 7 | 28.73 | 0.00 | 0.00 |
| GO:0051098 | regulation of binding | 4 | 135.42 | 0.00 | 0.00 |
| GO:0007183 | SMAD protein complex assembly | 4 | 67.71 | 0.00 | 0.00 |
| GO:0060395 | SMAD protein signal transduction | 6 | 13.11 | 0.00 | 0.00 |
| GO:0045216 | cell-cell junction organization | 4 | 30.09 | 0.00 | 0.00 |
| GO:0032909 | regulation of transforming growth factor beta2 production | 3 | 101.56 | 0.00 | 0.00 |
| GO:0017015 | regulation of transforming growth factor beta receptor signaling pathway | 4 | 27.08 | 0.00 | 0.01 |
| GO:0010862 | positive regulation of pathway-restricted SMAD protein phosphorylation | 5 | 14.11 | 0.00 | 0.01 |
| GO:0007179 | transforming growth factor beta receptor signaling pathway | 6 | 8.83 | 0.00 | 0.01 |
| GO:0001701 | in utero embryonic development | 7 | 5.07 | 0.00 | 0.03 |
| GO:0060391 | positive regulation of SMAD protein import into nucleus | 3 | 31.25 | 0.00 | 0.04 |
| GO:0010718 | positive regulation of epithelial to mesenchymal transition | 7 | 28.73 | 0.00 | 0.00 |
| GO:0001666 | response to hypoxia | 9 | 7.09 | 0.00 | 0.00 |
| GO:0060395 | SMAD protein signal transduction | 6 | 13.11 | 0.00 | 0.00 |
| GO:0045216 | cell-cell junction organization | 4 | 30.09 | 0.00 | 0.00 |
| GO:0032909 | regulation of transforming growth factor beta2 production | 3 | 101.56 | 0.00 | 0.00 |
| GO:0010862 | positive regulation of pathway-restricted SMAD protein phosphorylation | 5 | 14.11 | 0.00 | 0.01 |
| GO:0010936 | negative regulation of macrophage cytokine production | 3 | 81.25 | 0.00 | 0.01 |
| GO:0007179 | transforming growth factor beta receptor signaling pathway | 6 | 8.83 | 0.00 | 0.01 |
| GO:0007435 | salivary gland morphogenesis | 3 | 58.04 | 0.00 | 0.01 |
| GO:0007184 | SMAD protein import into nucleus | 3 | 45.14 | 0.00 | 0.02 |
| GO:0048839 | inner ear development | 4 | 13.21 | 0.00 | 0.04 |
| GO:0006283 | transcription-coupled nucleotide-excision repair | 5 | 9.15 | 0.00 | 0.03 |
| GO:1904837 | beta-catenin-TCF complex assembly | 4 | 12.60 | 0.00 | 0.04 |
| GO:0000185 | activation of MAPKKK activity | 3 | 36.93 | 0.00 | 0.03 |
| GO:0043550 | regulation of lipid kinase activity | 3 | 81.25 | 0.00 | 0.01 |
| GO:1900034 | regulation of cellular response to heat | 6 | 10.83 | 0.00 | 0.00 |

Supplementary Table2. KEGG pathways of cell cycle genes

| Pathway ID | Pathway name | Gene count | Fold enrichment | P value | Benjamini-adjusted P value |
| --- | --- | --- | --- | --- | --- |
| hsa04914 | Progesterone-mediated oocyte maturation | 29 | 18.49 | 0.00 | 0.00 |
| hsa04120 | Ubiquitin mediated proteolysis | 19 | 7.69 | 0.00 | 0.00 |
| hsa05161 | Hepatitis B | 26 | 9.95 | 0.00 | 0.00 |
| hsa05220 | Chronic myeloid leukemia | 20 | 15.41 | 0.00 | 0.00 |
| hsa05212 | Pancreatic cancer | 15 | 12.80 | 0.00 | 0.00 |
| hsa05200 | Pathways in cancer | 31 | 4.38 | 0.00 | 0.00 |
| hsa05222 | Small cell lung cancer | 15 | 9.79 | 0.00 | 0.00 |
| hsa05215 | Prostate cancer | 15 | 9.46 | 0.00 | 0.00 |
| hsa05219 | Bladder cancer | 11 | 14.88 | 0.00 | 0.00 |
| hsa05206 | MicroRNAs in cancer | 22 | 4.27 | 0.00 | 0.00 |
| hsa05214 | Glioma | 11 | 9.39 | 0.00 | 0.00 |
| hsa05218 | Melanoma | 11 | 8.59 | 0.00 | 0.00 |
| hsa05223 | Non-small cell lung cancer | 9 | 8.92 | 0.00 | 0.00 |
| hsa05210 | Colorectal cancer | 10 | 8.95 | 0.00 | 0.00 |
| hsa04310 | Wnt signaling pathway | 12 | 4.82 | 0.00 | 0.00 |
| hsa04919 | Thyroid hormone signaling pathway | 9 | 4.34 | 0.00 | 0.00 |
| hsa05211 | Renal cell carcinoma | 6 | 5.04 | 0.01 | 0.02 |

Supplementary table3. Differentially expressed genes in the GEO dataset

| Gene | AveExpNormal | AveExpCancer | T value | P.Value |  | adj.P.Val |
| --- | --- | --- | --- | --- | --- | --- |
| *ABL1* | 5.90 | 6.22 | 6.54 | 0.00 |  | 0.00 |
| *ANAPC1* | 5.48 | 5.98 | 13.38 | 0.00 |  | 0.00 |
| *ANAPC10* | 4.50 | 4.67 | 3.77 | 0.00 |  | 0.02 |
| *ANAPC5* | 6.62 | 7.00 | 11.95 | 0.00 |  | 0.00 |
| *ATR* | 5.29 | 6.10 | 19.61 | 0.00 |  | 0.00 |
| *BUB1* | 3.41 | 3.63 | 13.09 | 0.00 |  | 0.00 |
| *BUB1B* | 3.80 | 5.58 | 24.32 | 0.00 |  | 0.00 |
| *BUB3* | 6.85 | 7.77 | 20.53 | 0.00 |  | 0.00 |
| *CCNA1* | 3.66 | 3.55 | 5.50 | 0.00 |  | 0.00 |
| *CCNA2* | 3.60 | 4.69 | 18.83 | 0.00 |  | 0.00 |
| *CCNB1* | 3.62 | 6.10 | 30.80 | 0.00 |  | 0.00 |
| *CCNB2* | 3.95 | 5.41 | 22.76 | 0.00 |  | 0.00 |
| *CCND1* | 7.17 | 6.54 | 6.55 | 0.00 |  | 0.00 |
| *CCNE1* | 3.72 | 4.47 | 11.85 | 0.00 |  | 0.00 |
| *CCNE2* | 3.39 | 4.28 | 17.59 | 0.00 |  | 0.00 |
| *CDC14B* | 5.87 | 5.02 | 19.03 | 0.00 |  | 0.00 |
| *CDC16* | 6.93 | 7.19 | 5.47 | 0.00 |  | 0.00 |
| *CDC20* | 3.68 | 5.61 | 23.99 | 0.00 |  | 0.00 |
| *CDC23* | 5.54 | 6.56 | 20.84 | 0.00 |  | 0.00 |
| *CDC25A* | 3.81 | 4.12 | 11.24 | 0.00 |  | 0.00 |
| *CDC25B* | 6.12 | 7.03 | 14.23 | 0.00 |  | 0.00 |
| *CDC25C* | 3.53 | 3.78 | 13.44 | 0.00 |  | 0.00 |
| *CDC27* | 5.07 | 5.61 | 18.18 | 0.00 |  | 0.00 |
| *CDC45* | 3.75 | 4.29 | 14.70 | 0.00 |  | 0.00 |
| *CDC6* | 3.48 | 4.34 | 17.05 | 0.00 |  | 0.00 |
| *CDC7* | 3.77 | 4.88 | 17.39 | 0.00 |  | 0.00 |
| *CDK1* | 3.69 | 5.44 | 25.73 | 0.00 |  | 0.00 |
| *CDK2* | 4.10 | 4.27 | 7.71 | 0.00 |  | 0.00 |
| *CDK4* | 6.50 | 7.77 | 21.65 | 0.00 |  | 0.00 |
| *CDK6* | 3.75 | 3.63 | 5.23 | 0.00 |  | 0.00 |
| *CDK7* | 4.96 | 6.02 | 17.68 | 0.00 |  | 0.00 |
| *CDKN1A* | 6.98 | 6.61 | 4.70 | 0.00 |  | 0.00 |
| *CDKN2A* | 3.85 | 4.41 | 12.64 | 0.00 |  | 0.00 |
| *CDKN2C* | 4.62 | 5.53 | 19.14 | 0.00 |  | 0.00 |
| *CHEK1* | 3.28 | 3.79 | 13.78 | 0.00 |  | 0.00 |
| *CHEK2* | 4.18 | 4.85 | 12.99 | 0.00 |  | 0.00 |
| *CUL1* | 6.69 | 7.01 | 7.19 | 0.00 |  | 0.00 |
| *DBF4* | 4.15 | 5.42 | 22.78 | 0.00 |  | 0.00 |
| *E2F1* | 4.13 | 4.33 | 7.04 | 0.00 |  | 0.00 |
| *E2F2* | 3.51 | 3.43 | 4.12 | 0.00 |  | 0.01 |
| *E2F3* | 4.61 | 5.46 | 19.25 | 0.00 |  | 0.00 |
| *E2F4* | 5.58 | 5.44 | 5.15 | 0.00 |  | 0.00 |
| *E2F5* | 3.52 | 4.15 | 11.21 | 0.00 |  | 0.00 |
| *EP300* | 5.09 | 5.33 | 5.47 | 0.00 |  | 0.00 |
| *ESPL1* | 5.04 | 5.62 | 9.92 | 0.00 |  | 0.00 |
| *GADD45A* | 9.97 | 8.43 | 20.00 | 0.00 |  | 0.00 |
| *GADD45B* | 6.95 | 5.75 | 18.60 | 0.00 |  | 0.00 |
| *GADD45G* | 5.57 | 4.57 | 12.42 | 0.00 |  | 0.00 |
| *GSK3B* | 6.34 | 6.50 | 3.61 | 0.00 |  | 0.04 |
| *HDAC1* | 8.17 | 8.75 | 12.48 | 0.00 |  | 0.00 |
| *HDAC2* | 7.24 | 8.08 | 15.79 | 0.00 |  | 0.00 |
| *MAD1L1* | 5.67 | 5.07 | 8.54 | 0.00 |  | 0.00 |
| *MAD2L1* | 3.41 | 4.92 | 21.01 | 0.00 |  | 0.00 |
| *MCM2* | 4.27 | 5.93 | 22.76 | 0.00 |  | 0.00 |
| *MCM3* | 5.33 | 6.92 | 24.75 | 0.00 |  | 0.00 |
| *MCM4* | 4.09 | 5.06 | 21.70 | 0.00 |  | 0.00 |
| *MCM5* | 4.37 | 5.53 | 21.77 | 0.00 |  | 0.00 |
| *MCM6* | 5.29 | 7.37 | 26.00 | 0.00 |  | 0.00 |
| *MCM7* | 5.33 | 6.53 | 22.04 | 0.00 |  | 0.00 |
| *MDM2* | 4.10 | 3.95 | 6.46 | 0.00 |  | 0.00 |
| *ORC1* | 3.51 | 3.69 | 6.38 | 0.00 |  | 0.00 |
| *ORC3* | 5.79 | 6.36 | 12.52 | 0.00 |  | 0.00 |
| *ORC4* | 5.22 | 5.50 | 9.44 | 0.00 |  | 0.00 |
| *ORC5* | 4.19 | 4.45 | 10.47 | 0.00 |  | 0.00 |
| *ORC6* | 5.90 | 6.48 | 11.12 | 0.00 |  | 0.00 |
| *PCNA* | 7.24 | 8.61 | 20.61 | 0.00 |  | 0.00 |
| *PKMYT1* | 4.15 | 4.30 | 4.90 | 0.00 |  | 0.00 |
| *PLK1* | 3.68 | 4.03 | 11.78 | 0.00 |  | 0.00 |
| *PRKDC* | 4.42 | 5.34 | 21.89 | 0.00 |  | 0.00 |
| *PTTG1* | 5.32 | 7.82 | 34.64 | 0.00 |  | 0.00 |
| *RAD21* | 6.88 | 8.13 | 20.89 | 0.00 |  | 0.00 |
| *RB1* | 4.72 | 4.85 | 3.79 | 0.00 |  | 0.02 |
| *RBL1* | 3.51 | 3.78 | 8.35 | 0.00 |  | 0.00 |
| *RBL2* | 7.46 | 6.45 | 18.70 | 0.00 |  | 0.00 |
| *RBX1* | 7.45 | 8.14 | 17.07 | 0.00 |  | 0.00 |
| *SFN* | 4.73 | 6.39 | 14.62 | 0.00 |  | 0.00 |
| *SKP1* | 8.53 | 9.00 | 10.70 | 0.00 |  | 0.00 |
| *SKP2* | 5.03 | 5.29 | 6.88 | 0.00 |  | 0.00 |
| *SMAD2* | 5.08 | 5.94 | 19.38 | 0.00 |  | 0.00 |
| *SMAD3* | 4.48 | 4.68 | 8.60 | 0.00 |  | 0.00 |
| *SMC1A* | 5.56 | 5.88 | 11.70 | 0.00 |  | 0.00 |
| *SMC3* | 5.44 | 6.39 | 14.14 | 0.00 |  | 0.00 |
| *STAG1* | 5.19 | 5.82 | 16.27 | 0.00 |  | 0.00 |
| *STAG2* | 6.95 | 7.39 | 9.43 | 0.00 |  | 0.00 |
| *TFDP1* | 4.97 | 5.51 | 8.88 | 0.00 |  | 0.00 |
| *TFDP2* | 4.61 | 4.84 | 6.38 | 0.00 |  | 0.00 |
| *TP53* | 4.29 | 4.55 | 6.26 | 0.00 |  | 0.00 |
| *TTK* | 3.35 | 5.04 | 22.69 | 0.00 |  | 0.00 |
| *YWHAB* | 8.31 | 8.97 | 15.98 | 0.00 |  | 0.00 |
| *YWHAH* | 5.65 | 6.79 | 17.75 | 0.00 |  | 0.00 |
| *YWHAQ* | 8.82 | 9.54 | 15.78 | 0.00 |  | 0.00 |
| *YWHAZ* | 6.97 | 7.98 | 17.34 | 0.00 |  | 0.00 |

Supplementary table4. Differentially expressed genes in the TCGA dataset

| Gene | AveExpNormal | AveExpCancer | T value | P.Value | adj.P.Val |
| --- | --- | --- | --- | --- | --- |
| *CDK4* | 1253.77 | 1983.14 | -6.11 | 0.00 | 0.00 |
| *RBL1* | 25.36 | 73.11 | -4.27 | 0.00 | 0.01 |
| *RBL2* | 2352.47 | 1539.12 | 5.43 | 0.00 | 0.00 |
| *E2F1* | 41.45 | 574.55 | -5.38 | 0.00 | 0.00 |
| *E2F2* | 9.22 | 67.77 | -5.53 | 0.00 | 0.00 |
| *E2F4* | 585.19 | 816.50 | -4.92 | 0.00 | 0.00 |
| *E2F5* | 45.23 | 111.69 | -4.62 | 0.00 | 0.00 |
| *GSK3B* | 839.97 | 1050.34 | -3.98 | 0.00 | 0.02 |
| *SMAD2* | 866.51 | 1249.18 | -4.28 | 0.00 | 0.01 |
| *MYC* | 3267.25 | 1620.22 | 4.36 | 0.00 | 0.00 |
| *ZBTB17* | 363.72 | 481.09 | -5.06 | 0.00 | 0.00 |
| *CDKN2A* | 21.66 | 302.79 | -6.07 | 0.00 | 0.00 |
| *CDKN2B* | 90.71 | 217.74 | -5.80 | 0.00 | 0.00 |
| *CDKN2C* | 99.42 | 518.22 | -5.24 | 0.00 | 0.00 |
| *CDC6* | 33.57 | 307.67 | -6.19 | 0.00 | 0.00 |
| *CDC45* | 12.20 | 132.76 | -7.38 | 0.00 | 0.00 |
| *CDC7* | 24.39 | 105.09 | -4.43 | 0.00 | 0.01 |
| *DBF4* | 74.86 | 137.69 | -6.20 | 0.00 | 0.00 |
| *CDK1* | 33.10 | 358.98 | -6.00 | 0.00 | 0.00 |
| *CCNB1* | 57.69 | 495.29 | -8.63 | 0.00 | 0.00 |
| *CCNB2* | 15.63 | 219.02 | -7.74 | 0.00 | 0.00 |
| *CCNB3* | 13.20 | 23.66 | -4.69 | 0.00 | 0.00 |
| *CDC25B* | 725.96 | 1387.39 | -5.18 | 0.00 | 0.00 |
| *CDC25C* | 3.83 | 112.65 | -5.95 | 0.00 | 0.00 |
| *YWHAZ* | 5986.60 | 9505.88 | -4.63 | 0.00 | 0.00 |
| *YWHAB* | 3776.75 | 4794.83 | -5.02 | 0.00 | 0.00 |
| *YWHAH* | 1869.57 | 2539.96 | -3.80 | 0.00 | 0.03 |
| *YWHAG* | 3307.78 | 4337.52 | -3.80 | 0.00 | 0.04 |
| *PLK1* | 21.65 | 284.63 | -7.08 | 0.00 | 0.00 |
| *WEE1* | 1841.84 | 817.55 | 5.38 | 0.00 | 0.00 |
| *PKMYT1* | 15.55 | 163.52 | -7.06 | 0.00 | 0.00 |
| *CCNH* | 703.57 | 521.23 | 4.86 | 0.00 | 0.00 |
| *CDK7* | 257.82 | 350.33 | -4.25 | 0.00 | 0.01 |
| *ANAPC1* | 268.22 | 399.02 | -4.20 | 0.00 | 0.01 |
| *ANAPC4* | 257.23 | 363.16 | -5.23 | 0.00 | 0.00 |
| *ANAPC5* | 2052.69 | 2476.47 | -5.03 | 0.00 | 0.00 |
| *ANAPC7* | 529.28 | 919.73 | -10.21 | 0.00 | 0.00 |
| *CDC23* | 558.52 | 835.55 | -6.29 | 0.00 | 0.00 |
| *CDC20* | 22.08 | 404.90 | -6.11 | 0.00 | 0.00 |
| *PTTG1* | 28.24 | 362.93 | -6.64 | 0.00 | 0.00 |
| *SMC1A* | 1705.57 | 2355.66 | -4.23 | 0.00 | 0.01 |
| *RAD21* | 2375.18 | 3799.92 | -5.54 | 0.00 | 0.00 |
| *TTK* | 5.57 | 112.19 | -5.64 | 0.00 | 0.00 |
| *BUB1* | 11.34 | 191.69 | -6.87 | 0.00 | 0.00 |
| *BUB3* | 1104.83 | 1476.88 | -5.52 | 0.00 | 0.00 |
| *BUB1B* | 11.45 | 150.59 | -6.91 | 0.00 | 0.00 |
| *MAD2L1* | 41.13 | 212.23 | -6.00 | 0.00 | 0.00 |
| *CDC14B* | 1475.02 | 928.37 | 4.45 | 0.00 | 0.00 |
| *ATR* | 307.01 | 460.40 | -4.93 | 0.00 | 0.00 |
| *CHEK1* | 50.59 | 139.64 | -6.41 | 0.00 | 0.00 |
| *PRKDC* | 1531.12 | 2848.03 | -5.58 | 0.00 | 0.00 |
| *GADD45A* | 3409.99 | 2001.97 | 4.75 | 0.00 | 0.00 |
| *GADD45B* | 12543.03 | 3665.02 | 6.58 | 0.00 | 0.00 |
| *GADD45G* | 4754.48 | 1427.66 | 6.47 | 0.00 | 0.00 |
| *PCNA* | 915.82 | 1743.68 | -6.08 | 0.00 | 0.00 |
| *SFN* | 55.33 | 932.39 | -4.46 | 0.00 | 0.01 |
| *CDC25A* | 22.84 | 94.38 | -5.97 | 0.00 | 0.00 |
| *MCM2* | 165.47 | 882.24 | -6.39 | 0.00 | 0.00 |
| *MCM3* | 663.59 | 1735.69 | -7.11 | 0.00 | 0.00 |
| *MCM4* | 293.56 | 843.98 | -5.60 | 0.00 | 0.00 |
| *MCM5* | 588.55 | 1530.07 | -6.15 | 0.00 | 0.00 |
| *MCM6* | 254.29 | 872.62 | -6.41 | 0.00 | 0.00 |
| *MCM7* | 841.59 | 1973.80 | -8.32 | 0.00 | 0.00 |

Supplementary table5. The area under curve(AUC) values of 52 differentially expressed genes in HCC

| Gene | AUC (GEO) | AUC(TCGA) |
| --- | --- | --- |
| *CDC14B* | 87.68 | 82.88 |
| *GADD45A* | 89.85 | 78.40 |
| *GADD45B* | 87.10 | 88.44 |
| *GADD45G* | 79.61 | 86.68 |
| *RBL2* | 88.47 | 78.84 |
| *ANAPC1* | 80.76 | 71.32 |
| *ANAPC5* | 79.54 | 79.60 |
| *ATR* | 89.62 | 74.76 |
| *BUB1* | 84.99 | 96.92 |
| *BUB1B* | 95.98 | 94.92 |
| *BUB3* | 90.15 | 81.12 |
| *CCNB1* | 98.08 | 96.84 |
| *CCNB2* | 93.68 | 95.12 |
| *CDC20* | 95.39 | 96.76 |
| *CDC23* | 90.29 | 81.92 |
| *CDC25A* | 76.61 | 87.00 |
| *CDC25B* | 82.13 | 74.00 |
| *CDC25C* | 81.74 | 97.04 |
| *CDC45* | 83.68 | 96.12 |
| *CDC6* | 91.86 | 95.04 |
| *CDC7* | 89.68 | 85.56 |
| *CDK1* | 96.50 | 96.40 |
| *CDK4* | 92.45 | 86.76 |
| *CDK7* | 86.47 | 70.60 |
| *CDKN2A* | 79.63 | 92.92 |
| *CDKN2C* | 89.41 | 93.20 |
| *CHEK1* | 86.78 | 88.00 |
| *DBF4* | 95.14 | 82.16 |
| *E2F1* | 67.95 | 96.52 |
| *E2F5* | 78.32 | 75.28 |
| *GSK3B* | 60.29 | 74.76 |
| *MAD2L1* | 95.09 | 92.56 |
| *MCM2* | 93.23 | 92.48 |
| *MCM3* | 95.06 | 93.48 |
| *MCM4* | 94.50 | 85.72 |
| *MCM5* | 92.29 | 88.40 |
| *MCM6* | 94.17 | 94.16 |
| *MCM7* | 93.68 | 90.88 |
| *PCNA* | 92.26 | 82.92 |
| *PKMYT1* | 62.40 | 97.68 |
| *PLK1* | 77.85 | 97.00 |
| *PRKDC* | 92.84 | 81.04 |
| *PTTG1* | 98.16 | 96.72 |
| *RAD21* | 91.30 | 80.92 |
| *RBL1* | 71.77 | 76.08 |
| *SFN* | 81.19 | 79.20 |
| *SMAD2* | 88.7362 | 76.28 |
| *SMC1A* | 79.8226 | 70.96 |
| *TTK* | 95.21394 | 95.32 |
| *YWHAB* | 87.58043 | 76.68 |
| *YWHAH* | 87.51491 | 68.76 |
| *YWHAZ* | 86.51284 | 73.08 |

Supplementary Table6. The associations of 35 cell cycle genes with overall survival of HCC patients

| Gene | Kaplan-Meier survival analysis | | Univariate analysis | | | | |
| --- | --- | --- | --- | --- | --- | --- | --- |
|  | Chisq value | P value | Median | OR | 2.5%CI | 97.5%CI | P value |
| *RBL2* | 4.45 | 0.03 | 6.45 | 0.62 | 0.36 | 1.03 | 0.07 |
| *ABL1* | 9.80 | 0.00 | 6.22 | 2.16 | 1.28 | 3.68 | 0.00 |
| *HDAC1* | 4.77 | 0.03 | 8.80 | 1.63 | 0.97 | 2.74 | 0.07 |
| *HDAC2* | 6.77 | 0.01 | 8.04 | 1.63 | 0.97 | 2.74 | 0.07 |
| *E2F3* | 6.04 | 0.01 | 5.41 | 1.74 | 1.04 | 2.95 | 0.04 |
| *GSK3B* | 7.64 | 0.01 | 6.49 | 0.46 | 0.27 | 0.78 | 0.00 |
| *TGFB2* | 6.20 | 0.01 | 3.25 | 1.87 | 1.11 | 3.17 | 0.02 |
| *CDKN2B* | 9.79 | 0.00 | 3.61 | 2.57 | 1.52 | 4.40 | 0.00 |
| *CDKN1B* | 4.44 | 0.04 | 7.71 | 0.62 | 0.36 | 1.03 | 0.07 |
| *CDKN1C* | 6.62 | 0.01 | 3.99 | 1.87 | 1.11 | 3.17 | 0.02 |
| *SKP2* | 4.61 | 0.03 | 5.24 | 1.63 | 0.97 | 2.74 | 0.07 |
| *CCNA2* | 4.39 | 0.04 | 4.51 | 1.52 | 0.90 | 2.55 | 0.12 |
| *CDK1* | 11.15 | 0.00 | 5.35 | 2.16 | 1.28 | 3.68 | 0.00 |
| *CDC25B* | 4.05 | 0.04 | 7.07 | 1.52 | 0.90 | 2.55 | 0.12 |
| *PLK1* | 3.94 | 0.05 | 3.94 | 1.52 | 0.90 | 2.55 | 0.12 |
| *WEE1* | 5.56 | 0.02 | 6.43 | 1.63 | 0.97 | 2.74 | 0.07 |
| *CDC20* | 8.76 | 0.00 | 5.52 | 1.74 | 1.04 | 2.95 | 0.04 |
| *PTTG2* | 3.93 | 0.05 | 3.22 | 1.82 | 1.08 | 3.07 | 0.02 |
| *SMC3* | 7.80 | 0.01 | 6.47 | 1.87 | 1.11 | 3.17 | 0.02 |
| *STAG1* | 6.03 | 0.01 | 5.79 | 1.74 | 1.04 | 2.95 | 0.04 |
| *RAD21* | 9.89 | 0.00 | 8.12 | 2.32 | 1.38 | 3.96 | 0.00 |
| *TTK* | 6.31 | 0.01 | 4.93 | 1.52 | 0.90 | 2.55 | 0.12 |
| *BUB3* | 6.07 | 0.01 | 7.80 | 1.87 | 1.11 | 3.17 | 0.02 |
| *MAD2L1* | 5.71 | 0.02 | 4.77 | 1.63 | 0.97 | 2.74 | 0.07 |
| *CDC14B* | 6.56 | 0.01 | 4.94 | 0.57 | 0.34 | 0.96 | 0.04 |
| *CHEK1* | 6.72 | 0.01 | 3.63 | 1.87 | 1.11 | 3.17 | 0.02 |
| *GADD45A* | 6.19 | 0.01 | 8.44 | 0.62 | 0.36 | 1.03 | 0.07 |
| *CDC25A* | 8.72 | 0.00 | 4.07 | 2.01 | 1.20 | 3.41 | 0.01 |
| *ORC6* | 8.62 | 0.00 | 6.29 | 2.01 | 1.20 | 3.41 | 0.01 |
| *MCM2* | 9.49 | 0.00 | 5.90 | 2.01 | 1.20 | 3.41 | 0.01 |
| *MCM3* | 8.26 | 0.00 | 6.86 | 1.87 | 1.11 | 3.17 | 0.02 |
| *MCM4* | 5.97 | 0.01 | 4.99 | 1.74 | 1.04 | 2.95 | 0.04 |
| *MCM5* | 12.36 | 0.00 | 5.49 | 2.32 | 1.38 | 3.96 | 0.00 |
| *MCM6* | 9.57 | 0.00 | 7.50 | 2.01 | 1.20 | 3.41 | 0.01 |
| *MCM7* | 6.43 | 0.01 | 6.44 | 1.74 | 1.04 | 2.95 | 0.04 |

Supplementary Table7. The associations of 12 cell cycle genes with recurrence-free survival of HCC patients in the GEO dataset

|  | Kaplan-Meier recurrence analysis | |  | | Univariate analysis | |  | |  | Multivariate analysis | | | |
| --- | --- | --- | --- | --- | --- | --- | --- | --- | --- | --- | --- | --- | --- |
|  | Chisq value | P value | OR | 2.50%CI | | 97.50%CI | | P value | | OR | 2.50%CI | 97.50%CI | P value |
| *CDKN1C* | 4.12 | 0.04 | 1.50 | 0.90 | | 2.50 | | 0.12 | | 1.37 | 0.79 | 2.36 | 0.26 |
| *CDC25B* | 4.73 | 0.03 | 1.40 | 0.84 | | 2.34 | | 0.20 | | 1.39 | 0.81 | 2.40 | 0.24 |
| *CDC20* | 6.07 | 0.01 | 1.31 | 0.79 | | 2.18 | | 0.30 | | 0.98 | 0.56 | 1.71 | 0.95 |
| *PTTG2* | 6.65 | 0.01 | 2.15 | 1.29 | | 3.64 | | 0.00 | | 2.17 | 1.24 | 3.86 | 0.01 |
| *SMC3* | 4.10 | 0.04 | 1.31 | 0.79 | | 2.18 | | 0.30 | | 1.17 | 0.67 | 2.04 | 0.59 |
| *RAD21* | 7.87 | 0.01 | 1.84 | 1.10 | | 3.09 | | 0.02 | | 1.88 | 1.08 | 3.28 | 0.03 |
| *MAD1L1* | 3.93 | 0.05 | 0.58 | 0.35 | | 0.97 | | 0.04 | | 0.53 | 0.30 | 0.93 | 0.03 |
| *EP300* | 4.19 | 0.04 | 1.50 | 0.90 | | 2.50 | | 0.12 | | 1.49 | 0.85 | 2.63 | 0.16 |
| *GADD45A* | 5.83 | 0.02 | 0.71 | 0.43 | | 1.19 | | 0.20 | | 0.76 | 0.44 | 1.32 | 0.33 |
| *GADD45G* | 3.87 | 0.05 | 0.67 | 0.40 | | 1.11 | | 0.12 | | 0.62 | 0.35 | 1.07 | 0.09 |
| *CDC25A* | 4.59 | 0.03 | 1.40 | 0.84 | | 2.34 | | 0.20 | | 1.29 | 0.74 | 2.27 | 0.37 |
| *MCM5* | 4.48 | 0.03 | 1.50 | 0.90 | | 2.50 | | 0.12 | | 1.26 | 0.73 | 2.19 | 0.40 |

Supplementary Table8. The associations of 12 cell cycle genes with recurrence-free survival of HCC patients in the TCGA dataset

|  | Kaplan-Meier recurrence analysis | | Univariate analysis | | | | |
| --- | --- | --- | --- | --- | --- | --- | --- |
| Gene | Chisq value | P value | Median | OR | 2.50%CI | 97.50%CI | P value |
| *CDKN1C* | 0.73 | 0.39 | 70.09 | 0.92 | 0.58 | 1.45 | 0.71 |
| *CDC25B* | 3.10 | 0.08 | 1199.01 | 1.27 | 0.80 | 2.02 | 0.30 |
| *CDC20* | 8.06 | 0.00 | 294.94 | 1.72 | 1.09 | 2.74 | 0.02 |
| *PTTG2* | 0.06 | 0.81 | 0.59 | 1.11 | 0.70 | 1.76 | 0.65 |
| *SMC3* | 0.73 | 0.39 | 903.35 | 1.21 | 0.77 | 1.92 | 0.41 |
| *RAD21* | 0.62 | 0.43 | 3534.80 | 0.93 | 0.59 | 1.47 | 0.75 |
| *MAD1L1* | 0.01 | 0.92 | 545.36 | 1.20 | 0.76 | 1.90 | 0.43 |
| *EP300* | 0.95 | 0.33 | 1288.25 | 0.88 | 0.55 | 1.39 | 0.58 |
| *GADD45A* | 5.21 | 0.02 | 1435.36 | 1.00 | 0.63 | 1.59 | 0.99 |
| *GADD45G* | 2.54 | 0.11 | 1132.27 | 0.94 | 0.60 | 1.49 | 0.80 |
| *CDC25A* | 6.12 | 0.01 | 75.51 | 1.39 | 0.88 | 2.20 | 0.16 |
| *MCM5* | 4.56 | 0.03 | 1333.61 | 1.24 | 0.79 | 1.97 | 0.35 |

Supplementary Figure1. The Kaplan-Meier survival analysis results of *BUB3*(A), *CDK1*(B), *CHEK1*(C) in the TCGA dataset.
